# The Enrolment Scheme and the Profession

**Published:** 1916-04-22

**Authors:** 


### The Hospital

The Workers' Newspaper of Administrative Medicine and Institutional Life, Administration, National Insurance and Health.

No. 1558, Vol. LX. SATURDAY, APRIL 22, 1916. PRICE ONE PENNY

### THE ENROLMENT SCHEME AND THE PROFESSION.

The statement, which is also an appeal, recently issued by the War Office, places a final and authoritative approval on the scheme of enrolment formulated by the Central Medical War Committee for England and Wales, and by the kindred Committees relating to Scotland and Ireland respectively. The claim is that all medical practitioners whose age does not exceed forty-five years should formally indicate their willingness to take service with the Army if in the opinion of the War Office and of the War Committee such service is required. There is not the slightest anticipation that all such practitioners will be called upon to accept commissions. On the contrary, the great majority in all probability will be asked to continue the civil practices in which they are at present engaged. But the time has arrived in which it is urgently necessary, in order to meet both the wants of the Army and the needs of the civil population, that the medical profession be organised in such a fashion that practitioners shall be distributed on a definite and effective plan. This necessity, be it observed, is imposed as much by the requirements of the civil population as by those of the Army. Unless there is such a plan chaos and confusion are likely to reign supreme. As the authorities call for more military medical officers—and the call will come without question—volunteers would doubtless be forthcoming, for our fighting men cannot be left without medical aid and direction. But the response in reference to individual districts, unless there is some central controlling authority and organisation, would more than likely be unequal and irregular, with the necessary consequence that while in some areas there would be a sufficiency of doctors for civil practice, in others the supply would be reduced to a perilous level. If such a result is to be avoided it is impossible to leave the choice of volunteering to individual initiative. There are doctors enough—but not too many—both for the needs of the Army and for the requirements of civil practice. But they must be distributed according to some organised scheme and systematic plan, and such a condition implies a central controlling authority. This, in a few words, is the position in which the profession stands in relation to the nation and the national cause. The question has passed beyond the limits of individual choice and individual convenience. It has become an issue of corporate responsibility, and the profession is summoned to organise itself in order that this responsibility may be adequately and honourably discharged.

The demand is certainly one involving the sacrifice of individual interests. Most of the men to whom the appeal for individual enrolment comes have entered on a career which cannot be interrupted without some risk of personal loss and professional prejudice. They are building up a livelihood on a plan which requires continuity for anything like full success. To leave their homes and their work is neither to their inclination nor to their interests. Yet it is only by an affirmative answer on their part that the profession can justify itself in the face of the nation. The claim of the profession has always been to serve the nation. It is now called upon to justify this claim in face of a great emergency in which the success and even the life of the country are at stake, and to think of failure in such circumstances is out of the question. Part of the demand is that the individual practitioner shall put out of his own hands the choice of his particular sphere of duty. He must yield his own will and judgment to those of others, and no one will dispute that this is a hard thing to ask of men who are conscious of many personal obligations and who have reached an age in which liberty of decision is in ordinary circumstances a full right. The reply, of course, is that we no longer live in ordinary circumstances. The days are big with fate, and the practitioner must yield to the patriot. It is duty to the nation which stands in the forefront, and medical practitioners, as others, must listen to the summons, even though in doing so there is a sacrifice of the opportunity for free choice and a risk of personal loss. All that can be said in mitigation of such a position is, first, that it is imposed on the nation; and secondly, that the solution of each individual's problem will be undertaken by a Committee which has no aim save the common good, and which is widely representative of all the interests concerned. This is the apparatus by which each man's sphere of duty will be fixed, and he must be content to abide the issue. His own judgment and views and circumstances may be fully stated and will be carefully considered in relation both to the position of his immediate colleagues and to the necessities of the situation. But the final word must be left to others. This, we repeat, is a hard thing to ask. But it is inevitable in the circumstances if the profession is to vindicate its position in the national life and to embrace a great opportunity for national service. Both within and without the profession public opinion, we believe, will now without hesitation endorse the enrolment scheme as the only method by which the wants of the country and the Army can be satisfactorily met.

Our expectation is that the scheme will be a large success. To make it so it must receive the practically unanimous support of practitioners of military age, or at least the dissentients must be a "negligible quantity." The Central Committee will not proceed to call up any practitioners unless and until 75 per cent. of the practitioners concerned have entered their names. Thus those who stand aside announce their determination to follow their own interests and refuse a professional judgment in preference to their own. In this way they will help to wreck a plan which has for its aim the maximum utility of the profession at a time of great national strain. Individual judgment and personal decision are high qualities when personal issues have to be adjudged. But there are times when these qualities must yield to larger interests, and the present moment comes well within this category.

